# Multi-Metaomics Unveils the Development Process of Microbial Communities During the Fermentation of Baobaoqu

**DOI:** 10.3390/foods14213657

**Published:** 2025-10-27

**Authors:** Qingchun Luo, Xi Li, Jianghua Li, Yanping Lu, Jian Chen, Jian Su, Dong Zhao, Jiao Hu, Xia Zhang, Pengju Zhao, Zhu Zhang, Qingmei Zhang, Xuejun Lei, Jinhe Bai, Jia Zheng, Xinrui Zhao

**Affiliations:** 1 Science Center for Future Foods, Jiangnan University, 1800 Lihu Road, Wuxi 214122, China; 7220210001@stu.jiangnan.edu.cn (Q.L.); lijianghua@jiangnan.edu.cn (J.L.); jchen@jiangnan.edu.cn (J.C.); 2 Key Laboratory of Industrial Biotechnology, Ministry of Education, School of Biotechnology, Jiangnan University, 1800 Lihu Road, Wuxi 214122, China; 3 Jiangsu Province Engineering Research Center of Food Synthetic Biotechnology, Jiangnan University, 1800 Lihu Road, Wuxi 214122, China; 4 Engineering Research Center of Ministry of Education on Food Synthetic Biotechnology, Jiangnan University, 1800 Lihu Road, Wuxi 214122, China; 5 Wuliangye Yibin Co., Ltd., Yibin 644000, China; 1710307128@pku.edu.cn (X.L.); luyanpingscut@163.com (Y.L.); sujian@wuliangye.com.cn (J.S.); zhaodong@wuliangye.com.cn (D.Z.); 17317814614@163.com (J.H.); zx860610@126.com (X.Z.); zhaopj19@tsinghua.org.cn (P.Z.); zhangzhucd@163.com (Z.Z.); zqm_zoe@163.com (Q.Z.); leixuejun333@sina.com (X.L.); 18811179952@163.com (J.B.); 6 Key Laboratory of Wuliangye-Flavor Liquor Solid-State Fermentation, China National Light Industry, Yibin 644000, China; 7 Solid-State Fermentation Resource Utilization Key Laboratory of Sichuan Province, Yibin 644007, China

**Keywords:** Baobaoqu, microbial community, enzymes, metagenomics, metatranscriptomics, metaproteomics, metabolic pathways

## Abstract

In order to understand the dynamic interaction process among species, enzymes, and metabolites during the fermentation process of Baobaoqu, which is a representative Daqu starter for Chinese baijiu, the intimate connection between the progression of microbial communities and the diversities and activities of enzymes was examined by metagenomics, metatranscriptomics and metaproteomics. It was found that while 5211 species of microorganisms were detected by metagenomics, only 1774 active species were detected by metatranscriptomics, which indicated that only a small proportion (34.04%) were active. The metabolic routes associated with the breakdown of substrates and synthesis of metabolites were redesigned, and the special functional microorganisms for lactate, pyrazines and phenylethyl alcohol production were isolated. It was found that the progression of the microbial community was highly coupled with the components of enzymes and flavor substrates, precisely corresponding to the three stages of the Baobaoqu fermentation process, and were regulated by multiple physical factors. During the Baobaoqu-making process of the fermentation, microorganisms with different functions work together to complete metabolism in different stages. These findings will aid us in gaining a deeper and clearer understanding of the “species–enzyme–metabolite” system within the Daqu starter culture, thus offering valuable perspectives for developing artificial synthetic communities and the production of high-quality Baobaoqu.

## 1. Introduction

Baijiu is a traditional alcoholic drink with a legacy that extends over millennia. As one of the six internationally acclaimed distilled spirits, it features alcohol concentrations between 35% and 60% [1]. According to its flavor characteristics, Baijiu can be categorized into three primary varieties: Nong-flavor, Jiang-flavor, and Qing-flavor. Of these, Nong-flavor baijiu comprises half of the sales market, attributed to its exceptional aromatic profile, smooth texture, and lingering finish [2]. Wuliangye, the quintessential brand of Nong-flavor baijiu, boasts a brewing heritage that exceeds 4000 years and ranks among the most favored alcoholic drinks in China. Renowned for its distinctive aroma and flavor characteristics, Wuliangye holds a significant place in the history of the Chinese baijiu industry. Its fermentation techniques have been extensively embraced, solidifying its status as one of the most pivotal baijiu brands in China.

**Figure 1:**
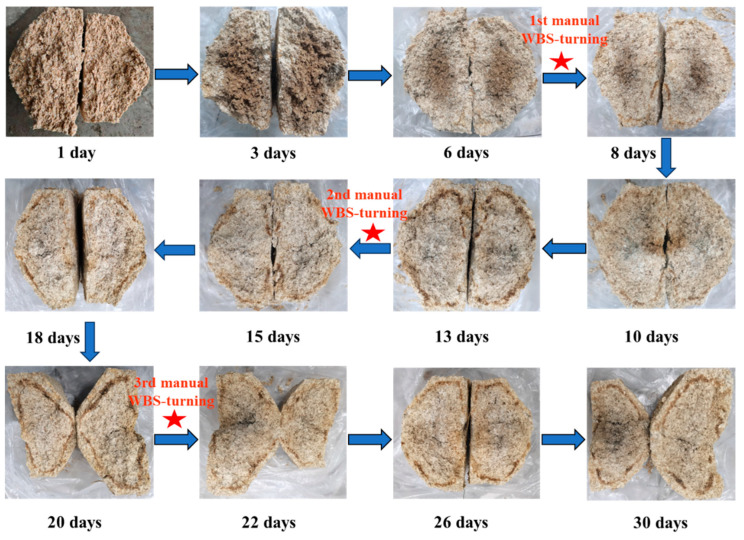
Variation in Baobaoqu during the fermentation process containing the cross-sectional diagrams of the Baobaoqu at 12 key time points (WBS: Wuliangye Baobaoqu starter).

Baijiu production utilizes an open fermentation system where a diverse array of microbes from the environment, raw ingredients, fermentation pits, and Daqu plays an essential role in the fermentation process of Baijiu [3]. Daqu is a crucial fermentation agent, composed of a mixture of enzymes and microorganisms. Based on the highest temperature reached during its production, Daqu is classified into three types: medium-temperature variety (40–50 °C), medium–high-temperature variety (50–60 °C), and high-temperature variety (60–70 °C). The Baobaoqu used to produce Wuliangye Baijiu is a typical representation of medium–high-temperature Daqu. Unlike other medium–high-temperature Daqu, Baobaoqu is made entirely from pure wheat [4]. Before the Daqu-making process begins, raw wheat is milled and water is added to achieve a moisture content of 38%. The wheat flour is then compressed into brick-shaped fermentation agents with protrusions on one side (Figure 1). These are then placed in a fermentation chamber for approximately 30 days. During this period, the temperature of the Baobaoqu rises from the ambient temperature to a peak level (58–60 °C), with the first manual repositioning occurring on the 7th day, marking the warming stage or Stage 1. Following this stage, the starter is maintained at peak temperature for an additional week under controlled conditions. The second manual re-positioning takes place on the 14th day, initiating the high-temperature phase, or stage 2. By the conclusion of this phase, most of the moisture content has evaporated, and the starter’s temperature gradually decreases. The third manual re-positioning occurs on the 21st day, after which the starters are consolidated to facilitate further moisture evaporation until the incubation process concludes, known as the temperature-fall phase, or stage 3. During this time, many microorganisms are cultivated and various essential enzymes and aroma compounds are formed, which determine the quantity and quality of Baijiu [4].

In recent years, the swift progress in omics technology has facilitated numerous studies focusing on the enzyme composition and microbial communities present in Daqu [5,6]. Zhang et al. analyzed the fermentation starter using a combination of metagenomics and metabolomics and compared the variations in microbial community composition and flavor constituents among varied medium–high-temperature variety Daqu starters [2]. Huang and colleagues [7] examined the distinctions in the characteristics of enzymes, the composition of microorganisms, and the functions of metabolism among three types of Daqu by employing both high-throughput sequencing and metagenomic techniques. However, microbes detected by metagenomics include all active and inactive microbes [8]. The components that actually play roles are the active microorganisms. Working out how to conduct targeted research on these active microorganisms has become a challenge [9]. Notably, metatranscriptomics has been widely employed as a powerful method to pinpoint active functional groups within the microbial communities of diverse fermented foods and MT (medium-temperature) Daqu [10]. Furthermore, metaproteomics, which is a powerful method to detect the complex protein composition in different fermentation materials, has been used to study the expressional levels of enzymes in MHT (medium–high-temperature) [11] and HT (high-temperature) Daqu starters [12]. Recently, data-independent acquisition mass spectrometry (DIA-MS) has proven its effectiveness in analyzing complex metaproteomic samples [13]. Specifically, the newest library-free DIA data analysis techniques [14], attain comprehensive proteome coverage and consistent quantification without relying on pre-established spectral libraries [15,16], showing great potential in the analysis of metaproteome profiling for large sample cohorts. The development of “omics” technology makes it possible to examine and integrate observed characteristics at various biological levels, encompassing DNA, RNA, proteins, and metabolites. These techniques enable us to make novel biological discoveries. To date, some microorganisms and enzymes of starters have been reported by metagenomics, metatranscriptomics, and metaproteomics, respectively. However, none of these reports have comprehensively examined and integrated the observed characteristics of MHT Daqu from multiple biological dimensions, such as the genome, transcripts, and metabolites.

This study aims to investigate the active microbial community, enzymes, and aroma compounds in Baobaoqu fermentation using multi-omics approaches such as metagenomics, metatranscriptomics, and metaproteomics [2,4,11]. We reconstructed metabolic pathways related to substrate decomposition and compound production to identify key microorganisms and their functions. Additionally, we pinpointed functional species associated with enzymatic activities and isolated those responsible for flavor compound production. These findings enhance our understanding of the microbial community’s structure and functional properties in Baobaoqu, providing a foundation for selecting strains with biological potential and guiding the acquisition of characteristic functional enzymes. Ultimately, this research will deepen our understanding of the interactions within the “species–enzymes–metabolite” system during Baobaoqu production, offering valuable insights for future synthetic community development. It is impossible to conduct a comprehensive study of microorganisms, enzymes, flavor, and metabolic networks simultaneously through a single omics approach.

## 2. Materials and Methods

### 2.1. Sample Collection

All Baobaoqu samples were collected from Wuliangye Co., Ltd., located in Yibin City, Sichuan Province, China, in June to July 2023. samples were collected on 1, 3, 6, 8, 10, 13, 15, 18, 20, 22, 26, and 30 days during the fermentation of Baobaoqu. At each sampling time point, the samples were obtained from three distinct rooms, with each sample being prepared by randomly crushing and blending three Baobaoqu bricks from same room. A total of 108 Baobaoqu bricks were chosen to create 36 representative samples, which were then split into two portions for subsequent analyses: one for assessing physicochemical parameters, conducting microbial isolation and culture tests, and determining flavor substances (stored at 4 °C), and the other for multi-omics analysis (stored at −80 °C).

### 2.2. Determination of Physicochemical and Enzymatic Parameters

The physicochemical properties included moisture, acidity and temperature. The moisture and acidity characteristics of Baobaoqu were evaluated according to the established procedures specified in the Light Industry Standard of the People’s Republic of China, QB/T 4257-2011. The moisture level was assessed using a gravimetric technique, which involved heating at 105 °C for 4 h [10], The acidity was evaluated with a pH meter following a 30 min extraction process, using a mixture of Baobaoqu powder and CO_2_-free water in a 1:10 ratio [7]. The core temperature of the Baobaoqu brick was measured using a stainless-steel thermometer. The enzymatic characteristics of Baobaoqu, which include saccharification, fermentation, liquefaction, and esterification capabilities, were evaluated following the procedures detailed in the Light Industry Standard of the People’s Republic of China, QB/T 4257-2011 [17]. The esterification capacity indicates the proficiency in forming esters from free organic acids and ethanol. In summary, 25 g (based on dry content) of Baobaoqu powder were introduced into a 250 mL conical flask that contained 25 mL of anhydrous ethanol, 1.5 mL of caproic acid, and 75 mL of distilled water. At the same time, blank experiments were done. 25 mL sterile water was introduced into a 250 mL conical flask that contained 25 mL of anhydrous ethanol, 1.5 mL of caproic acid, and 75 mL of distilled water. The mixture was then incubated at 35 °C for 7 days to facilitate esterification [7]. The cultivated blend was filtered using a membrane with a pore size of 0.2 μm, and the ethyl caproate content was measured using headspace solid-phase microextraction combined with gas chromatography-mass spectrometry (HS-SPME-GC-MS). Finally, the esterification power can be calculated. All samples were measured in triplicate.

### 2.3. Analysis of Volatile Compounds by HS-SPME/GC–MS

The volatile compounds were analyzed using HS-SPME-GC-MS, following the methodology outlined by Qingchun Luo et al., with three replicates conducted for each sample [18]. In summary, a sample weighing 3.0 g was combined with 10 mL of saturated NaCl solution in a headspace vial, along with 7.5 μL of 4-octanol solution (407 mg/L, used as an internal standard).

Volatile compounds were obtained by employing an 80 μm DVB/CAR/PDMS fiber at a temperature of 60 °C for 30 min. Analysis was conducted using GC-MS, which was outfitted with a DB-WAX column measuring 60 m in length, 0.32 mm in internal diameter, and having a film thickness of 0.25 μm. The carrier gas used was ultrahigh-purity helium, which was kept at a consistent flow rate of 1.0 mL per minute. The oven started at a temperature of 40 °C, where it remained for 5 min before being heated at a rate of 5 °C per minute until it reached 230 °C. This final temperature was held constant for 10 min. Volatile compounds were identified and analyzed semi-quantitatively based on a previously established method [18]. We used the NIST (national institute of standards and technology) library 20.L for compound identification. When determining the content of flavor components, an internal standard sample was added, 4-octanol, with a concentration of 407 ppm. Based on the peak areas of the flavor components and the peak areas of 4-octanol, the concentration of the flavor components was calculated.

The relative levels of volatile aroma compounds in Baobaoqu were assessed by evaluating the ratios of peak areas between the 4-octanol solution and the aromatic compounds. To explore the variations in metabolites among different Baobaoqu samples, Orthogonal Partial Least Squares Discriminant Analysis (OPLS-DA) was conducted utilizing SIMCA-14.1 software (Umetrics, Sweden). Metabolites with a VIP (Variable Importance for the Projection) score exceeding 1.0 were categorized as differential metabolites.

### 2.4. Total DNA, RNA and Protein Extraction

A 500 mL beaker was used to combine 50 g of the Baobaoqu sample, 250 mL of PBS buffer, and 10 glass beads, and this mixture was vigorously shaken for 30 min. Subsequently, the samples underwent centrifugation at 1500 rpm for 5 min, and the supernatants were collected into a centrifuge tube. Next, 5 mL of PBS buffer was added to the pellet for washing, a process that was repeated twice. The pellets were then centrifuged again at 1500 rpm for 5 min, and the resulting supernatants were collected. Spin the obtained supernatant at 12,000 rpm for a duration of 10 min, and collect the bacterial pellets. The collected samples were used for total genomic DNA extraction.

Following the manufacturer’s guidelines, the MagBeads FastDNA kit for soil (MP Biomedicals, Irvine, CA, USA) and the Rapid RNA Micro Extraction Kit (Gentone Biotech, Ningbo, China) were employed to extract total genomic DNA and RNA from Baobaoqu samples after they had been ground with liquid nitrogen. The extracted DNA and RNA were then stored at −80 °C until further analysis. The DNA and RNA concentrations and their purity levels were measured with a NanoDrop ND1000 spectrophotometer (Thermo Scientific, Waltham, MA, USA).

The method of total protein extraction was used as previously mentioned [11], and appropriate optimization was made. The main optimization extraction operation was as follows: 2 g of Baobaoqu and 12 mL of protein extraction solution was added, and the mixture was vigorously ground with liquid nitrogen for 10 min. Then, 12 mL of Tris saturated phenol solution was added and the mixture was ground for another 10 min. The upper 25 mL of the solution was collected, and the sample was centrifuged at 6500 rpm in a centrifuge tube for 10 min. The protein-containing solution was taken, and the remaining steps were the same as those in Reference 11. Each sample was performed in triplicate biological replicates.

### 2.5. DNA and RNA Library Construction and Sequencing, Enzymatic Hydrolysis and LC-MA/MS Analysis of Protein

The DNA extracted in the previous phase was initially fragmented into 300 bp segments through enzymatic digestion (RM0434, BGI Wuhan Biotechnology, Wuhan, China). For the construction of the DNA library, the fragmented DNA underwent end-repair, adapter ligation, and amplification via PCR utilizing the PMseq kit (BGI-Shenzhen, Shenzhen, China), in accordance with the guidelines provided by the manufacturer. DNA library was established. Subsequently, a universal kit was used for sequencing reaction on the MGISEQ-2000 platform (BGI-Shenzhen, China).

For metatranscriptomic analysis, following quality assessment and quantification, cDNA synthesis, and cDNA library construction in accordance with the manufacturer’s guidelines. The cDNA library was then sequenced on the Illumina HiSeq 4000 platform (BGI-Shenzhen, China). The initial metatranscriptomic sequences were subjected to thorough quality assessment, including the removal of the reads containing sequencing linker sequences, and low-quality reads (Q-score < 20), and environmental contaminants. The high-quality clean reads from each 36 Baobaoqu sample were assembled using the IDBA software (version 1.1.3) [19].

For the metaproteomics analysis, the obtained protein was mixed with an appropriate proportion of trypsin (1:40), and then was digested at 37 °C for 4 h. The peptides obtained were purified with a Strata X column to remove salts and subsequently dried using a vacuum process. For the purpose of DIA analysis using nano-LC-MS/MS, the peptide of each Baobaoqu were subjected to a C18 column (inner diameter 75 μm and length 25 cm) at the flow rate of 300 nL/min. The output results of the liquid chromatography process are directly interfaced with a mass spectrometer, and detection is performed under specified conditions: the peptides, having been separated via liquid chromatography, are ionized using the Captive Spray source and subsequently introduced into the timsTOF Pro2 tandem mass spectrometer for analysis using the DIA mode (BGI-Shenzhen, China).

### 2.6. Bioinformatics Analysis for Sequencing Data

Raw sequencing data of metagenomic and metatranscriptomic analyses underwent a multi-step preprocessing pipeline. For the analysis of metatranscriptomics, all raw datasets were processed and trimmed using SOAPnuke version 2.2.1 [20]. The processed reads were aligned with the host genome through SOAP2 software [21] to detect and eliminate sequences originating from the host. Subsequently, the refined metagenomic and metatranscriptomic datasets were assembled into contigs de novo using MEGAHIT [22] and Trinity (https://github.com/trinityrnaseq/trinityrnaseq/wiki/, accessed on 24 January 2024), respectively. Contigs with a length < 300 bp were excluded from the subsequent analysis.

MetaGeneMark was utilized to predict genes [23]. CD-HIT [24] was employed to eliminate redundant genes, applying a 95% identity threshold and a 90% coverage cutoff. The annotation details were produced by aligning the protein sequences of genes with functional databases such as BacMet, KEGG, EggNOG, COG, Swiss-Prot, and CAZy [25]. This alignment was performed using DIAMOND [26], applying an E-value threshold of 1 × 10^−5^. The taxonomic classification was determined utilizing the Kraken LCA algorithm [27].

To create profiles detailing taxonomic and functional abundance, the Bracken (https://github.com/jenniferlu717/Bracken, accessed on 24 January 2024) software is used with the default setting. Utilizing abundance profiles, the features such as Genera, Phyla, and KOs that exhibited significantly different abundances among groups were identified through the application of Wilcoxon’s rank sum test [28]. KEGG pathways with differential enrichment were discovered by analyzing reporter scores [29].

For metaproteomics analysis, the data were first performed with X!Tandem as iterative database search engine. For the analysis and quality control of DIA data, the mProphet algorithm was utilized, yielding numerous dependable quantitative outcomes. This process could also complete the GO, COG, Pathway, eggNOG, CAZy annotation analysis and species taxonomy analysis.

### 2.7. Statistical Analysis and Data Visualization

To evaluate significant differences among groups, we employed the Kruskal–Wallis test, deeming *p*-values under 0.05 as suggestive of statistical relevance. All statistical evaluations were performed utilizing the R programming platform. Additionally, the LEfSe method was applied, with an LDA (Linear Discriminant Analysis) threshold score exceeding 3.0, to pinpoint microbial biomarkers with notable taxonomic and functional disparities across groups. Principal Component Analysis (PCA) was employed to investigate differences in the microbial, genetic profiles and volatile compounds across the Baobaoqu samples. For metaproteomics, the process preprocessed the data according to the predefined comparison group, and then performed the significance test based on the model. Thereafter, differential protein screening was performed based on the fold change ≥2 and Pvalue < 0.05 as the criteria for the significant difference. At the same time, the enrichment analysis is performed on the differential proteins.

### 2.8. Verification of the Research Results on the Macroomics of Baobaoqu

Based on the results of metagenomics analysis (metagenomics, metatranscriptomics and metaproteomics), the active indicator microorganisms and functional microorganisms in the Baobaoqu were revealed. So how to precisely obtain these key functional microorganisms? Through the reconstructed metabolic network and by establishing precise screening methods for the production of lactic acid, pyrazine and phenylethyl alcohol.

#### 2.8.1. Isolation and Screening of Functional Microorganisms for Lactic Acid Production

The aim was to isolate and select the functional microorganisms for lactic acid production from Baobaoqu samples. A simple and efficient screening method was developed. Each Baobaoqu sample, weighing 5 g and collected at intervals of 3, 6, 8, 10, 13, and 15 days during the fermentation process, was combined with 45 mL of physiological saline solution that also contained glass beads. These mixtures were then homogenized by shaking in an incubator at 200 rpm for 30 min at room temperature. The mixed suspensions were subjected to a series of dilutions using a sterilized physiological saline solution. Subsequently, 100 μL of the suitably diluted solution were evenly distributed onto MRS (De Man, Rogosa and Sharpe) agar plates, sourced from Beijing Land Bridge, China, with 2 g/L natamycin and 5 × 10^−4^ g/L bromocresol violet for inhibiting fungus and indicating acid production, respectively. The plates were placed in an anaerobic environment using a Don Whitley Scientific M35 system and incubated at 37 °C for a period of 3 days. The system was infused with a gas mixture composed of 93% nitrogen, 2% hydrogen, and 5% carbon dioxide. Distinct colonies, demonstrating varied phenotypic characteristics, were chosen and purified. All the isolates underwent testing for acid production in alignment with the criteria defined for lactic acid bacteria [30] and were subsequently stored at 4 °C.

#### 2.8.2. Isolation and Screening of Functional Microorganisms for Pyrazine Production

The aim was to isolate and select the functional microorganisms for pyrazine production from Baobaoqu samples. Ammonia is the precursor of pyrazine, and the production of ammonia is related to the activity of protease, so the microorganisms with the function of pyrazine production are screened by using high-yield protease as a screening marker. The strains with high protease production were screened by hydrolytic transparent circle method [31]. 100 μL of appropriate diluted solution was spread on to skim milk medium plate, incubated at 30 °C for 48 h. The bacterial colonies with large hydrolytic transparent ring were selected as the strains with high protease production, and the pyrazine content was subsequently determined by HS-SPME-GC-MS.

#### 2.8.3. Isolation and Screening of Functional Microorganisms for Phenylethyl Alcohol Production

The aim was to isolate and select the functional microorganisms for phenylethyl alcohol production from Baobaoqu samples. Based on the results from the correlation analysis examining the relationship between active microorganisms and flavor constituents, phenylethyl alcohol are the most critical flavor substances. In the synthetic pathway of phosphoenolpyruvate, benzyl alcohol dehydrogenase (EC:1.1.1.90) and phenylpyruvate decarboxylase (EC:4.1.1.-) have the highest content of transcriptional genes involved in phenylethyl alcohol production. The active microorganisms recorded by the transcriptional genes were *Bacillus subtilis* and *Saccharomyces cerevisiae*. Therefore, the isolation medium of them were designed, and the phenylethyl alcohol content were subsequently determined.

## 3. Results and Discussion

### 3.1. Metabolite Profiling During Fermentation of Baobaoqu

**Figure 2:**
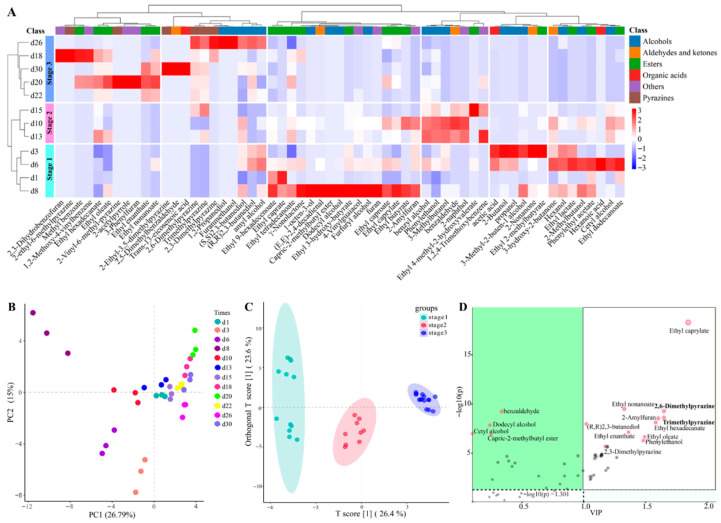
Variation in volatile compounds in the fermentation process of Baobaoqu by HS-SPME/GC-MS analysis, and the range of variation was −3 to +3 (**A**), PCA (principle component analysis) (**B**), OPLS-DA analysis (Orthogonal Partial Least Squares Discriminant Analysis) (**C**), and the results of difference correlation analysis between active microorganisms and volatile components (**D**).

The fermentation process and the ultimate quality of Baijiu are directly influenced by the quality of Daqu [32]. Consequently, a thorough assessment of Baobaoqu quality is essential, particularly the identification of its flavor compounds. In the fermentation of Baobaoqu, 60 distinct volatile compounds were detected (Table S1). The analysis of hierarchical clustering (Figure 2A) and PCA (Figure 2B) were performed to demonstrate distinct accumulation patterns of volatile compounds in the samples from different fermentation stages. As a result, the utilization of the supervised OPLS-DA model (Figure 2C) resulted in the effective separation of all volatile compounds [33].

During the initial phase of the temperature increase (day 1), there were fewer types and less content of volatile compounds. Between days 3 and 8, there was a significant accumulation of certain compounds, including higher alcohols such as 1-octen-3-ol, furfuryl alcohol, and 4-vinylguaiacol, organic acids like acetic acid and hexanoic acid, and ethyl esters such as ethyl caprate, ethyl caprylate, and ethyl dodecanoate. The acid-producing microorganisms contributed to the high production of acetic acid [34], which was consistent with the low pH value of 4.3. Then, during the high-temperature phase (days 10 to 15), enriched compounds included alcohols (e.g., benzyl alcohol and phenylethanol), and other aromatic compounds (e.g., 2-naphthol). In addition, the temperature-fall phase (days 18 to 30) was characterized by a dramatic increase within the levels of methyl esters, pyrazines, and various aromatic compounds. Especially, pyrazines, such as 2,3-dimethylpyrazine, were only detected in the temperature-fall phase. These results indicated that alcohols, esters and pyrazines were the main representative metabolites [35]. These findings suggest that metabolite accumulation varies distinctly across the different phases of Baobaoqu fermentation. Moreover, the development of flavor compounds is intimately linked to the microbial composition [36]. Consequently, understanding the evolution of microbial communities throughout the Baobaoqu fermentation process is of vital importance.

### 3.2. The Development Process of Microbial Community During Fermentation

**Figure 3:**
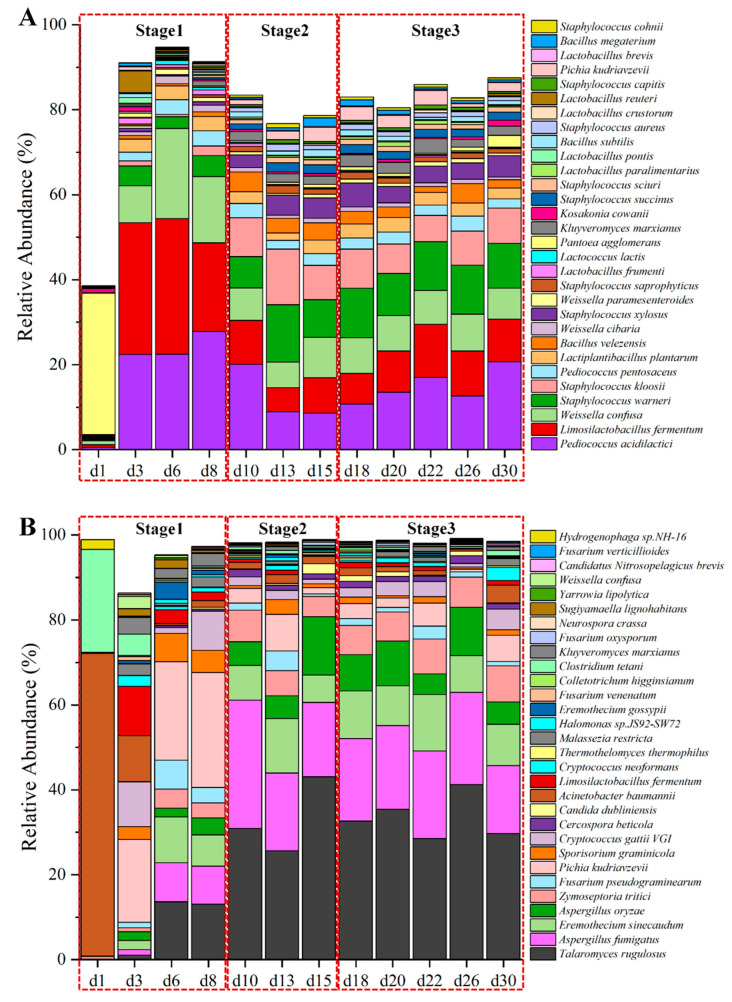
The absolute abundance variation in top 30 species dominant microorganisms by metagenomics (**A**) and active microorganisms by metatranscriptomics (**B**) at species level in the fermentation process of Baobaoqu.

A total of 2,768,338,688 reads were obtained by metagenomic sequencing, yielding 415.25 Gbp of raw data. Among the dataset, 357.10 Gbp of high-quality data were utilized for further analysis (refer to Table S2). Following the exclusion of wheat host sequences, the refined data were assembled into 5,327,925 unique contigs, achieving an N50 length of 2344 bp (Table S3). These contigs led to the prediction of 8,615,971 unigenes. In total, 205 orders (1468 genera and 5211 species) were identified, with the top ten most abundant orders (top 30 genera and top 30 species) representing over 98.70% (87.00% and 76.70%, respectively) of the relative abundance (Figure S1C,D), except on the initial day of fermentation (Figure 3A). On this first day, Enterobacterales, originating from wheat and the surrounding environment, predominated in the microbial community at the order level.

Along with the start of fermentation, when the temperature sharply rose, the *Lactobacillales* family quickly occupied the dominant role during the temperature-rise phase (days 3 to 8). The structure of microbial community was predominantly made up of *Limosilactobacillus* (*L. fermentum*), *Pediococcus* (*P. acidilactici*), and *Weissella* (*W. confusa*). According to the analysis of LEfSe (Figure S1E), these lactic acid bacteria containing *Lactiplantibacillus plantarum*, *Lactobacillus fermentum*, *Pediococcus acidilactici* and *Pediococcus pentosaceus* et al. were the biomarker species of Baobaoqu in the stage 1 (d3, d6 and d8) (LDA > 3), which producing lactic acid [2] and resulting in a lower pH value.

Subsequently, the structure of microbial community tended to stabilize from days 10 to 30. Once the rapid warming period ended, the structure of microbial community was predominantly occupied by *Staphylococcus* (*S. warneri*, *S. kloosii*), *Pediococcus* (*P. acidilactici*), *Limosilactobacillus* (*L. fermentum*), *Weissella* (*W. confusa*), *Pichia* (*P. kudriavzevii*, *Kluyveromyces* (*K. marxianus*), and *Bacillus* (*B. velezensis*), which accounted for approximately 80% of the total abundance (Figure 3A). The high-temperature resistant *Bacillus* and *Staphylococcus* species were the biomarker species of Baobaoqu in the stage 2, while *Pichia*, *Kluyveromyces*, *Saccharomycetales*, and *Aspergillus* species were the biomarker species of Baobaoqu in the stage 3. Numerous enzymes produced by thermophilic microorganisms have the ability to break down starches and proteins in raw grains, converting them into glucose and amino acids [37]. It has been noted that *Bacillus* species play a role in the development of aromatic compounds in Baijiu [37]. *Staphylococcus* species are capable of transforming nutrients like proteins and fats into volatile compounds, which contribute to a more complex and enhanced flavor profile. Moreover, these biomarker species can collaborate with other microorganisms such as lactic acid bacteria, yeast, and molds to jointly influence the formation of flavor [38]. These findings demonstrated that microorganisms engage in interactions at various stages to enhance flavor synthesis in Baobaoqu. Nonetheless, environmental conditions affected the growth patterns of these microorganisms, resulting in alterations in their physicochemical characteristics. Consequently, to investigate how environmental factors impact microbial growth, the study focused on assessing both physicochemical and enzymatic properties.

### 3.3. The Succession of Microorganisms Influenced by Temperature

**Figure 4:**
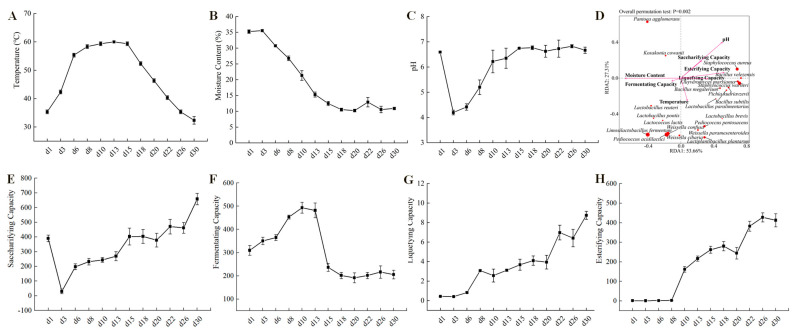
Dynamics of (**A**) temperature, (**B**) moisture content, (**C**) pH values, (**D**) RDA analysis (redundancy analysis) of the microbial and those physicochemical indicators, (**E**) saccharifying capacity, (**F**) fermenting capacity, (**G**) liquefying capacity, (**H**) esterifying capacity, during the Baobaoqu fermentation.

The key indicators for evaluating the quality of Baobaoqu are its physical and chemical characteristics, associated with the microbial functions in the starters [39]. The results of physicochemical and enzymatic properties are shown in Figure 4. Temperature plays a crucial role in the fermentation process of Baobaoqu, serving as a significant driving factor in shaping microbial communities. During the initial phase spanning from day 1 to day 8 (the temperature-rising stage), the temperature steadily climbed from room temperature, approximately 35 °C, to roughly 60 °C. Throughout this period, the moisture content remained relatively high at 26.8%. The suitable temperature and humidity created favorable conditions for the growth of lactic acid bacteria and other bacteria. According to the RDA analysis of microbial and physicochemical indicators of Baobaoqu (Figure 4D), the pH value was negatively correlated with the amount of lactic acid bacteria such as *L. fermentum*, *W. confusa*, and *P. acidilactici*., resulting in a significant decrease in pH value from 6.6 to 4.2 [34]. This suggests a direct correlation between the reduction in pH levels and the activity of these acid-producing lactic acid bacteria, aligning with earlier studies [34]. During the period from day 8 to day 15, known as the high-temperature phase, the temperature stabilized at approximately 59–60 °C (Figure 4A,B). During this period, the microbial community gradually evolved to heat-tolerant bacteria, such as *Staphylococcus* and *Bacillus*. This implies that temperature plays a pivotal role in the development of the microbial community throughout the fermentation of Baobaoqu [40]. During the cooling stage, the moisture content of Baobaoqu was 10.2–12.5% [41], which meets the industrial requirement (lower than 15%) [37,41], aligning with findings previously documented [42]. Once the fermentation process has concluded, the Daqu is stored for several days to reduce the moisture content to 15% and allow the temperature to return to room temperature [42]. During the cooling stage, the pH value fluctuated around 6.7 (Figure 4C). The findings indicated that temperature significantly influences the progression of microbial communities throughout Baobaoqu fermentation [10].

### 3.4. The Development Process of Active Microbial Community During Fermentation

#### 3.4.1. Active Species in Baobaoqu

The results of metagenomic analysis show that Baobaoqu contains a rich variety of microorganisms. To specifically explore the active microorganisms during fermentation, metatranscriptomics was applied. From 633,023,532 reads, we acquired a total of 385.41 Gbp of raw data, and after processing, 376.78 Gbp of clean data was extracted, representing 99.75% of the reads, for further analysis (Table S4). Following the elimination of host sequences from wheat, we obtained 854,736 unique contigs, achieving an N50 length of 870 base pairs (Table S4). Then, 947,720 unigenes were predicted.

In order to investigate the composition of active microbial communities throughout the fermentation process of Baobaoqu, we utilized alpha-diversity indices, specifically Chao1 and Shannon, to assess the species richness and diversity (Figure S2A,B). The findings revealed significant alterations in the diversity and richness of active microorganisms throughout the fermentation process. In particular, on the day 3 of fermentation, the Chao1 and Shannon index were significantly higher than at other time points during fermentation. PCA was employed to analyze the variations in the active microbial composition of Baobaoqu (Figure S2C). This analysis clearly demonstrated the distinct abundance of species from different fermentation stages. According to these results, the succession of active microbial compositions was obvious in stages 1 and 2 of fermentation and tended to stabilize after stage 3.

In total, 157 orders (723 genera and 1774 species) of active microorganisms were annotated in Baobaoqu. Of the 5211 species of microorganisms, only a small proportion were active (34.04%). The top ten dominant orders, along with the top 30 genera and top 30 species, comprised over 94.8% (95.7% and 86.3%) of the entire active microbial population, except for the first day of fermentation (Figure 3B). The findings revealed that the active microbial community was predominantly composed of fungi (>89.8%) after day 3 of fermentation, including Eurotiales, Saccharomycetales, Mycosphaerellales, Hypocreales, Tremellales, and Ustilaginales. The community also contained some bacteria, including Pseudomonadales, Lactobacillales, Bacillales, and Clostridiales (Figure S2D). At the level of genera, the dominant population (>5%) consisted of a total of 11 genera: *Talaromyces, Aspergillus, Zymoseptoria, Fusarium, Eremothecium, Cryptococcus, Sporisorium, Acinetobacter, Limosilactobacillus,* and *Clostridium* (Figure S2E).

As shown in Figure 3B, the top 30 predominant species of dominant active microorganisms in the fermentation of Baobaoqu differed in their relative abundance. Microorganisms exhibiting significant differences during the sequential fermentation periods of Baobaoqu were examined using LEfSe analysis, identifying those with an LDA score exceeding 3 as biomarker microorganisms. Among the active microorganisms identified, 21 species displayed a relative abundance of over 1%. According to the LEfSe analysis (Figure S2F), the marker bacterial genera of stage 1 (days 3 to 6) were *Limosilactobacillus*, *Pediococcus*, *Weissella*, *Kluyverromyces* and *Pichia*. *Aspergillus* (including *A. oryzae* and *A. fumigatus*), *Candida*, *Talaromyces* and *Cercospora* were the biomarkers of stage 2 (days 10 to 15) and stage 3. The acid-producing microorganisms, including *Limosilactobacillus*, *Pediococcus*, and *Weissella*, play a crucial role in supplying essential precursors for ester formation and facilitate the development of aromatic alcohols in Baijiu. In addition, *A. oryzae* can produce glucoamylase, which typically exhibits high hydrolytic activity towards the α-1,4 glucosidic bond [43]. This implies that variations in the relative abundance of these predominant active microorganisms will influence the ability of Baobaoqu to produce aroma.

#### 3.4.2. Functional Analysis of Baobaoqu

To gain insight into the roles of active microorganisms, the same KEGG pathways were annotated using metatranscriptomic analysis of 5688 KOs, which were generated from 40,916 unigenes. These pathways demonstrated a time-dependent characteristic, indicating that only certain microbial functions identified through metagenomics were persistently active. In the KEGG database, metabolic pathways are classified into 6 categories: Metabolism, Environmental Information Processing, Cellular Processes, Genetic Information Processing, Organismal Systems, and Human Diseases [44]. These pathways are further broken down into various sub-levels. This study calculated the abundance of secondary functional pathways within the KEGG database. The role of macromolecules in Baobaoqu microorganisms was ascertained by annotating sequencing outcomes and quantifying individual genes within the KEGG database. The findings revealed that metabolic pathways were the most prevalent. Among these, the relative abundances of pathways related to carbohydrate metabolism, amino acid metabolism, energy metabolism, lipid metabolism, cofactor and vitamin metabolism were high. As in previous reports [45], the amino acid metabolism and carbohydrate metabolism emerged as the prominent biological functions, which indicated that Baobaoqu has the functions of substrate hydrolysis and flavor generation.

#### 3.4.3. Enzymes in Baobaoqu

Based on the Enzyme Commission (EC) classification and metatranscriptomics findings, the 1532 identified enzymes can be categorized into seven distinct groups. The EC numbers of these 1532 enzymes, discovered in Baobaoqu, were sourced from Uniprot (www.uniprot.org) and are organized as follows: 324 oxidoreductases, 517 transferases, 345 hydrolases, 138 lyases, 84 isomerases, 92 ligases, and 32 translocases.

### 3.5. The Reconstructions of the Metabolic Pathways of Carbohydrates, Amino Acids, and Flavor Compounds

#### Metabolism of Carbohydrates

**Figure 5:**
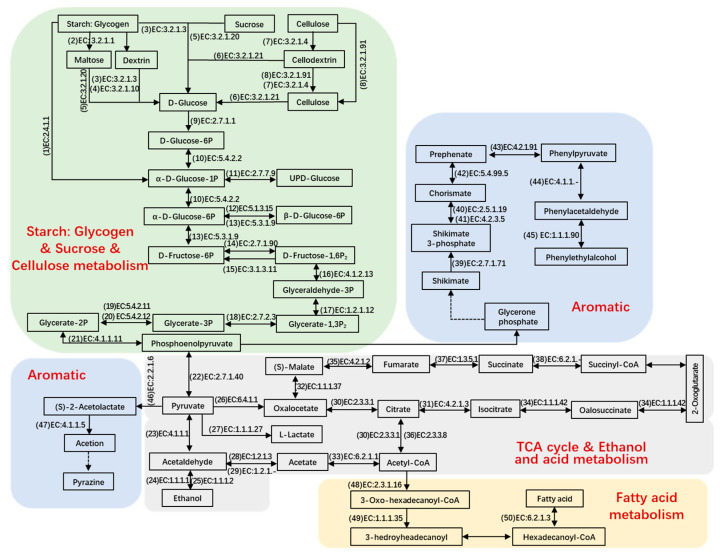
The reconstructed microbial metabolic networks related to the metabolism of carbohydrates and amino acids as well as the key enzymes and detected metabolites correlated with the pathways during fermentation based on KEGG and CAZy database in the Baobaoqu fermentation process.

To illustrate the key metabolic pathways responsible for transforming raw materials into final products, a number of metabolites were chosen for detailed analysis. Genes that actively code for a variety of carbohydrate-active enzymes and proteases facilitate the breakdown of polymers into monomers. During days 1 to 3, the vigorous breakdown of starch and cellulose resulted in the generation of pyruvate (Figure 5) [10]. Meanwhile, glycogen phosphorylase (EC number: 2.4.1.1), glucoamylase (EC number: 3.2.1.3), cellulase (EC number: 3.2.1.4), alpha-amylase (EC number: 3.2.1.1), alpha-glucosidase (EC number: 3.2.1.20), beta-glucosidase (EC number: 3.2.1.21), exoglucanase (EC number: 3.2.1.91), hexokinase (EC number: 2.7.1.1), phosphoglucomutase (EC number: 5.4.2.2), glucose-6-phosphate 1-epimerase (EC number: 5.1.3.15), phosphohexomutase (EC number: 5.3.1.9), diphosphate-dependent phosphofructokinase (EC number: 2.7.1.90), fructose-1,6-bisphosphatase (EC number: 3.1.3.11), glyceraldehyde-3-phosphate dehydrogenase (EC number: 1.2.1.12), fructose-bisphosphate aldolase (EC number: 4.1.2.13), phosphoglycerate kinase (EC number: 2.7.2.3), ambiguous phosphoglyceromutase (EC number: 5.4.2.11), ambiguous phosphoglyceromutase (EC number: 5.4.2.12), and ambiguous pyruvate kinase (EC number: 2.7.1.40), retained their elevated levels, the majority of which stemmed from *Saccharomyces*, *Aspergillus*, *Mycobacterium*, *Rhizomucor*, *Neosartorya*, *Thermobifida*, *Emericella*, *Lactobacillus*, and *Bacillus* (Figure S3). Furthermore, numerous hydrolytic enzymes demonstrated significant abundance starting from day 6, primarily produced by *Rhizopus*, *Aspergillus*, *Arabidopsis*, *Schizosaccharomyces*, and *Staphylococcus*. These enzymes likely represent the carbohydrate-active components in mature Baobaoqu.

When oxygen is insufficient, pyruvate may be transformed into lactate. The elevated presence of enzymes that metabolize lactate led to the substantial lactate content observed between days 3 and 8. The relevant enzyme was L-lactate dehydrogenase (EC number: 1.1.1.27), which was produced by *Lactobacillus* and *Weissella* and results in the low pH (4.2–5.2) in Baobaoqu. As for ethanol metabolism, the transformation of acetaldehyde into ethanol was facilitated by alcohol dehydrogenase (NADP^+^) (EC number: 1.1.1.2) and alcohol dehydrogenase (EC number: 1.1.1.1), with the former being highly expressed by *Schizosaccharomyces* and *Saccharomyces* on day 3 (Figure S3). As shown in Figure 4E, the esterification power rises in the stage 1. At the same time, the transcriptional level of pyruvate decarboxylase (EC number: 4.1.1.1), alcohol dehydrogenase and alcohol dehydrogenase (NADP^+^) also reached the peak level on the day 3.

The TCA cycle plays a pivotal role in numerous biochemical reactions, with nearly all of the ten key enzymes associated with the cycle being highly expressed from days 3 to 30. These enzymes include pyruvate carboxylase (EC 6.4.1.1), amino acid N-acetyltransferase (EC 2.3.1.1), aconitate hydratase (EC 4.2.1.3), acetyl CoA synthase (EC 6.2.1.1), malate dehydrogenase (EC 1.1.1.37), isocitrate dehydrogenase (EC 1.1.1.42), fumarate hydratase (EC 4.2.1.2), ATP citrate synthase (EC 2.3.3.8), succinate dehydrogenase (EC 1.3.5.1), and acyl-CoA synthetase (EC 6.2.1.-). The primary producers of these enzymes were identified as *Aspergillus*, *Staphylococcus*, *Bacillus*, *Saccharomyces*, *Schizosaccharomyces*, *Candida*, and *Yarrowia* (Figure S3).

It is crucial to illuminate the complex synthetic pathway of phenylethylalcohol for improving the quality of Baobaoqu. Shikimate appeared to be primarily transformed into chorismate and prephenate by the action of shikimate kinase (EC number: 2.7.1.71, originated from *Caldicellulosiruptor*, and *Lactobacillus*), (EC number: 2.5.1.19, 4.2.3.5 and 5.4.99.5, originated from *Leuconostoc*, *Neurospora*, and *Emericella*) after day 3, and then converted to phenylacetaldehyde by phenylpyruvate decarboxylase (EC number: 4.1.1.-, originated from *Bacillus*) after day 3. Phenylacetaldehyde can be converted into phenylethylalcohol by arylalcohol dehydrogenase (EC number: 1.1.1.90, originated from *Pseudomonas*). As the expression of arylalcohol dehydrogenase mainly occurred from day 3, the accumulation of phenylacetaldehyde also started from day 3. Then, phenylpyruvate decarboxylase was mainly expressed from day 10, resulting in the accumulation of phenylethylalcohol from days 10 to 15. These results were consistent with the flavor components in Baobaoqu.

In pyrazine metabolism, the transformation of pyruvate into (S)-2-acetolactate by acetolactate synthase is an irreversible reaction (EC number: 2.2.1.6) and is followed by the synthesis of acetoin catalyzed by acetolactate decarboxylase (EC number: 4.1.1.5). On the day 3, pyruvate was converted into more acidic substances, such as lactate and acetate. Therefore, despite the high expression of acetolactate synthase, the content of pyrazine substances was low in Baobaoqu. During stage 3 of fermentation (days 18–30), more pyruvate was converted into pyrazine, resulting in the higher level of 2, 6-dimethylpyrazine, trimethylpyrazine, 2, 3-dimethylpyrazine in the later stage.

In fatty acid metabolism, the related enzymes are acetyl-CoA acyltransferase (EC number: 2.3.1.16), 3-hydroxyacyl-CoA dehydrogenase (EC number: 1.1.1.35), and long-chain acyl-CoA synthetase (EC number: 6.2.1.3), which are originated from *Yarrowia*, *Mycobacterium*, *Arabidopsis*, and *Saccharomyces*.

### 3.6. The Profile of Proteins and Enzymes Analyzed by Metaproteomics

**Figure 6:**
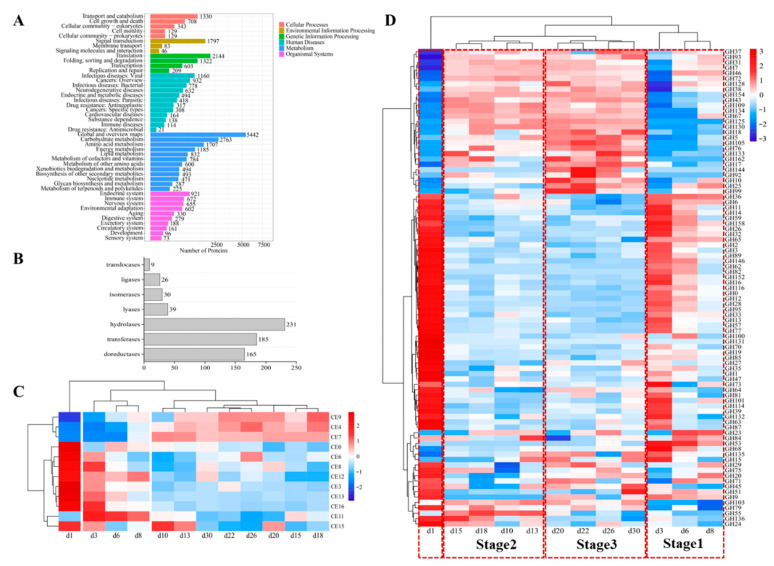
Histogram of pathway annotation and expressed enzymes by metaproteomics. (**A**) The *Y*-axis represents the KEGG functional classification entries and the *X*-axis represents the number of proteins in this functional classification. The metabolic pathways of different branches are distinguished by color. The KEGG metabolic pathway contains seven branches: Cellular Processes, Environmental Information Processing, Genetic Information Processing, Human Diseases, Metabolism, Organic Systems and Drug Development. (**B**) The vertical axis represents the CAZy function classification, and the horizontal axis represents the number of enzymes in the function classification. (**C**) The cluster analysis of the expressed carbohydrate esterase in the fermentation process of Baobaoqu, and the range of variation was −2 to +2. (**D**) The cluster analysis of the expressed glycoside hydrolases in the fermentation process of Baobaoqu, and the range of variation was −3 to +3.

To determine which enzymes were expressed, proteins were comprehensively isolated from the processed Baobaoqu, and digested into peptides by trypsin. The processed samples underwent analysis through label-free DIA metaproteomics, resulting in the identification and quantification of 19,293 proteins and 71,936 peptides across the 36 samples. To elucidate the functions of these proteins, they were classified into six distinct categories according to KEGG annotations. These categories encompassed cellular processes, environmental information processing, metabolism, genetic information processing, and organismal systems (Figure 6A). Notably, 45.51% of the total proteins were associated with metabolic activities, such as global and overview maps, amino acid metabolism, carbohydrate metabolism, among others. The 685 identified enzymes can be classified into seven subcategories according to the specific chemical reactions they catalyze, including 165 oxidoreductases, 185 transferases, 231 hydrolases, 39 lyases, 30 isomerases, 26 ligases and 9 translocase enzymes (Figure 6B).

To gain a more detailed understanding of the specific functions and potential applications of Baobaoqu, these enzymes were analyzed using the CAZy database. This analysis revealed that they belong to the category of glycoside hydrolases (GHs), which are capable of breaking glycosidic bonds. As a result, among these enzymes, hydrolase was the most abundant enzyme (Figure 6D) and belongs to 89 GH families. These GH families include GH12, GH135, GH152, GH28, GH31, GH33, GH47, GH6, GH70, and so on, which displayed the highest abundance and were related to carbohydrate hydrolysis. This result further supports the traditional view that Baobaoqu is a saccharifying starter culture [46]. The cluster analysis of these enzymes showed that, except for the samples from day 1, the hydrolases in Baobaoqu clustered into 3 classes (Figure 6D), which was consistent with the temperature stage (stage 1, 2, and 3). Specifically, in stage 1, GH6, GH3, GH65, and GH15 were highly expressed; in stage 2, GH5, GH7, GH10, GH10, and GH134 were highly expressed; in stage 3, GH18, GH10, GH17, and GH76 were highly expressed. Within the category of hydrolases, glucanase emerges as the predominant enzyme, followed by GH3 (beta-glucosidase, EC number: 3.2.1.21), endoglucanase (EC number: 3.2.1.4), GH15 (glucoamylase, EC number: 3.2.1.3), alpha-glucosidase (EC number: 3.2.1.20), and glucan 1,3-beta-glucosidase (EC number: 3.2.1.58). These findings align with the macro transcriptome results and the observed saccharification and liquefaction capabilities (Figure 4D,E). The dynamic succession and function of glycoside hydrolases during Baobaoqu fermentation are importance for the formation of flavors. This research revealed a strong correlation between the shifts in glycoside hydrolase diversity, the progression of microbial communities, and the alteration of flavor constituents. These changes are regulated by multiple factors, including temperature, humidity, and pH value [34].

As shown in Figure 6C, the carbohydrate esterase (CE) plays important roles in synthesizing esters and flavor substances in Baijiu. In this study, the related CE families were CE0, CE3, CE4, CE6, CE7, CE8, CE9, CE11, CE12, CE13, CE15, and CE16. Among these families, in stage 1, CE3, CE6, CE8, and CE13 were highly expressed. While in stage 2 and 3, CE4, CE7 and CE9 were highly expressed. In addition, as shown in Figure 4G, the esterification power increased after stage 2, which is associated with the higher expression of feruloyl esterase (EC number: 3.1.1.73), acetyl esterase (EC number: 3.1.1.-), pectin esterase (EC number: 3.1.1.11), and 6-phosphogluconolactonase (EC number: 3.1.1.31). It was found that *Staphylococcus* was the biomarker microorganism in stage 2, and the esterification power was significantly increased at this stage. Consistent with the previous report, a *S. epidermidis* YTPW-14 strain that produces high levels of esterase was isolated from Daqu [47].

### 3.7. Isolation and Screening of Functional Microorganisms in Baobaoqu

#### 3.7.1. Screening of Functional Microorganisms for Lactate Production

To verify the results of multi-metaomics analysis, the functional microorganisms in Baobaoqu were isolated and screened. Lactic acid, as a significant organic acid, greatly enhances the mouthfeel of Baijiu [48]. In the pyruvate metabolism pathway, L-lactate dehydrogenase (EC number: 1.1.1.27) exhibits the highest transcriptional level among the genes involved in lactic acid synthesis. Therefore, one of the key metabolic pathways of pyruvate involves its conversion into lactic acid. The active microorganisms identified by metatranscriptomics were *L. fermentum*, *P. acidilactici*, *L. acidophilus*, *L. salivarius*, and *W. confuse* (Figure S4A). However, the high expressional level of L-lactate dehydrogenase detected by metaproteomics were only in *L. fermentum* and *P. acidilactici* (Figure S4B). Therefore, we specifically designed an MRS medium for the separation and screening of lactic acid bacteria, which is used to isolate and obtain microorganisms that produce lactic acid.

To verify the accuracy of macrotranscriptome and metaproteomics results, lactic acid bacteria were isolated and screened from Baobaoqu. 8 lactic acid-producing microorganisms were isolated and were analyzed by 16S rRNA gene sequencing. Based on the BLAST+ 2.17.0 (https://blast.ncbi.nlm.nih.gov/Blast.cgi, accessed on 5 June 2024.) search of the 16S rRNA gene sequences showed that 5 strains exhibited the greatest genetic similarity to *L. fermentum* JCM 1173^T^ (99.9%), while 3 strains exhibited the greatest genetic similarity to *P. acidilactici* JCM 8797^T^ (99.8%), consistent with the expression of L-lactate dehydrogenase detected by metaproteomics. Furthermore, most of the *L. fermentum* and *P. acidilactici* were isolated from days 3 and 6 during the fermentation of Baobaoqu, which validated the results of transcriptome. Furthermore, the lactic acid content produced by the isolated strains was determined, revealing a maximum titer of 14.28 g/L.

#### 3.7.2. Screening of Functional Microorganisms for Pyrazines Production

The majority of pyrazines, such as trimethylpyrazine, 2,6-dimethylpyrazine, and tetramethylpyrazine, are characterized by aromas reminiscent of baked goods, nuts, and chocolate [4]. According to the difference correlation analysis for active microorganisms and flavor components (Figure 2D), 2,6-dimethylpyrazine and trimethylpyrazine are the most critical flavor substances. In the pyruvate metabolism pathway, the transcriptional genes involved in pyrazine production are most abundant in acetolactate synthase (EC number: 2.2.1.6) and acetolactate decarboxylase (EC number: 4.1.1.5). The active microorganisms identified by these genes are *Bacillus*, *Saccharomyces*, and *Lactococcus*. Therefore, we specifically designed an NA (Nutrient Agar) medium for the separation and screening of *Bacillus*, which is used to isolate and obtain microorganisms that produce pyrazines. In addition, 7 strains were screened in Baobaoqu for pyrazine production and *B. licheniformis* produced the highest titers of 2, 5-dimethylpyrazine (1.23 mg/L) and trimethylpyrazine (1.52 mg/L), respectively.

#### 3.7.3. Screening of Functional Microorganisms for Phenylethyl Alcohol Production

Phenylethylalcohol, with roselike aroma, is a by-product of yeast fermentation and a critical aroma compound in Baobaoqu based on GC/O analysis (Figure 2D) [4]. In the phosphoenolpyruvate metabolism pathway, the transcriptional genes involved in phenylethyl alcohol production are most abundant in phenylpyruvate decarboxylase (EC number: 4.1.1.-) and benzyl alcohol dehydrogenase (EC number: 1.1.1.90). The active microorganisms recorded by the transcriptional genes are *B. subtilis*, *S. cerevisiae*, and *P. putida*.

To verify the accuracy of macrotranscriptome results, microorganisms producing phenylethyl alcohol were isolated and screened from Baobaoqu. Therefore, we specifically designed a YPD (Yeast peptone dextrose) medium for the separation and screening of *S. cerevisiae*, which is used to isolate and obtain microorganisms that produce phenylethyl alcohol. Two yeasts were analyzed using 26S rRNA gene sequencing. A BLAST search showed that 2 strains were most closely to *S. cerevisiae* ATCC 18824^T^ (99.9%). The phenylethyl alcohol content producing by the isolated yeast was further determined, with the highest titer of 1.46 g/L.

The successful acquisition of lactate-, pyrazines-, and phenylethyl alcohol-producing functional strains proved the accuracy of macrotranscriptomics and metaproteomics in analyzing the functional microorganisms in Baobaoqu. It can be seen that the combined application of macro-omics technology has a certain theoretical guiding significance in screening functional microorganisms in Baobaoqu to enhance the quality of Baijiu.

## 4. Conclusions

In conclusion, this research sheds light on the biological significance and underlying mechanisms governing the progression of the active microbial community, as well as the composition of enzymes and aroma compounds in Baobaoqu fermentation, employing advanced multi-metaomics techniques such as metagenomics, metatranscriptomics, and metaproteomics. All of these cannot be achieved through a single type of omics technology alone. Our findings highlight a time-dependent cooperation among functional active microorganisms across various fermentation stages, revealing that the succession of the microbial community is intricately coupled with the diversity and activity of glycoside hydrolases and flavor components. These dynamics are regulated by multiple environmental factors, including temperature, humidity, and pH levels.

During the temperature-rise phase, starch-degrading bacteria play a critical role in converting macromolecules like starch into glucose, setting the stage for subsequent fermentation processes. As fermentation transitions into the high-temperature and temperature-fall phases, flavor-producing microorganisms emerge as key players in synthesizing important flavor compounds, such as pyrazine and phenylethyl alcohol, which contribute significantly to the sensory profile of Baijiu.

Our reconstruction of metabolic pathways related to substrate hydrolysis and metabolite development has elucidated the functional roles of and shifts in active microorganisms throughout the Baobaoqu fermentation process. By identifying species contributing to enzymatic activities through pathway analysis, we isolated microorganisms capable of producing lactate, pyrazines, and phenylethyl alcohol, subsequently confirming their production through quantitative analysis.

The biological implications of these discoveries extend beyond the realm of Baobaoqu, offering critical insights into the mechanisms of microbial community formation and function that can be applied to other fermented foods and industrial processes. For instance, similar multi-metaomics approaches can be utilized to optimize fermentation conditions in products such as yogurt, cheese, or vinegar, enhancing flavor profiles and nutritional content. Furthermore, understanding the dynamic “species–enzymes–metabolite” interactions in Baobaoqu fermentation may facilitate the design of artificial synthetic microbial communities tailored for specific industrial applications, leading to innovations in fermentation technology and bioprocessing. These insights will pave the way for future research aimed at enhancing the efficiency and consistency of fermentation processes across various sectors, ultimately contributing to the advancement of sustainable food production and industrial biotechnology.

## Data Availability

The original contributions presented in this study are included in the article; further inquiries can be directed to the corresponding author.

## Supplementary Materials

The following supporting information can be downloaded at: https://www.mdpi.com/article/10.3390/foods14213657/s1, Figure S1: The alpha-diversity indices (Chao1 and Shannon) analysis on the microbial species richness and diversity by metagenomic analysis (A,B). Variation of the microorganisms at orders (C) and genera (D) level in the fermentation process by metagenomic analysis. The investigation of biomarkers of the Baobaoqu by LEfSe analysis, with an LDA (Linear Discriminant Analysis) score exceeding 3 as biomarker microorganisms (E). Figure S2: The alpha-diversity indices (Chao1 and Shannon) analysis on the active microbial species richness and diversity by metatranscriptomics analysis (A,B). The principle component analysis (C). Variation of the active microorganisms at orders (D) and genera (E) level in the fermentation process by metatranscriptomics analysis. The investigation of biomarkers of the Baobaoqu by LEfSe analysis, with an LDA score exceeding 3 as biomarker microorganisms (F). Figure S3: Microbial sources (at the genus level) of key enzymes involved in the reconstructed microbial metabolic network in Figure 5. The Y axis depicts TPM abundances. Figure S4: Distribution map of active microorganisms derived from lactate dehydrogenase by by metatranscriptomics analysis (A). Microbial expression derived from lactate dehydrogenases by Metaproteomics analysis (B); Table S1: Summary of volatile compounds of Baobaoqu samples (ppm). Table S2: Raw and clean data statistics of Baobaoqu samples by metagenomics. Table S3: Assembly quality statistics of Baobaoqu samples by metagenomics. Table S4: Raw, clean data, and assembly quality statistics of Baobaoqu samples by metatranscriptomics.
