# Supplementary material for: Multi-Metaomics Unveils the Development Process of Microbial Communities During the Fermentation of Baobaoqu
Source: Foods. 2025 Oct 27;14(21):3657. doi: 10.3390/foods14213657 (PMC12610498; doi:10.3390/foods14213657)
Supplement: Supplementary file 1 [file foods-14-03657-s001.zip › foods-3932863-supplementary.pdf]

Article

Multi-metaomics unveils the development process of microbial communities during the fermentation of Baobaoqu

Qingchun Luo [1,2,3,4,5,7], Xi Li [5], Jianghua Li [1,2,3,4], Yanping Lu [5,6,7], Jian Chen [1,2,3,4], Jian Su [5,6,7], Dong Zhao [5,6,7], Jiao Hu [5], Xia Zhang [5], Pengju Zhao [5], Zhu Zhang [5], Qingmei Zhang [5], Xuejun Lei [5], Jinhe Bai [5], Jia Zheng [5,6,7,*], Xinrui Zhao [1,2,3,4,*]

[1] Science Center for Future Foods, Jiangnan University, 1800 Lihu Road, Wuxi, Jiangsu 214122, China;
[2] Key Laboratory of Industrial Biotechnology, Ministry of Education, School of Biotechnology, Jiangnan University, 1800 Lihu Road, Wuxi, Jiangsu 214122, China;
[3] Jiangsu Province Engineering Research Center of Food Synthetic Biotechnology, Jiangnan University, 1800 Lihu Road, Wuxi, Jiangsu 214122, China;
[4] Engineering Research Center of Ministry of Education on Food Synthetic Biotechnology, Jiangnan University, 1800 Lihu Road, Wuxi, Jiangsu 214122, China;
[5] Wuliangye Yibin Co., Ltd., Yibin, Sichuan 644000, China;
[6] Key Laboratory of Wuliangye-flavor Liquor Solid-state Fermentation, China National Light Industry, Yibin, Sichuan 644000, China;
[7] Solid-state Fermentation Resource Utilization Key Laboratory of Sichuan Province, Yibin 644007, China.
* Correspondence: author: zhengwanqi86@163.com; zhaoxinrui@jiangnan.edu.cn.

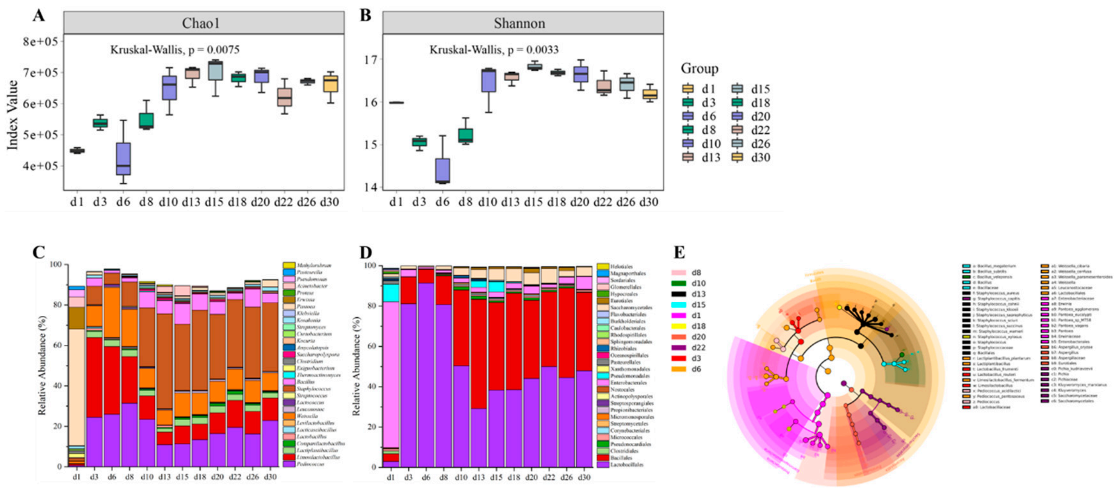

Figure S1. The alpha-diversity indices (Chao1 and Shannon) analysis on the microbial species richness and diversity by metagenomic analysis (A and B). Variation of the microorganisms at orders (C) and genera (D) level in the fermentation process by metagenomic analysis. The investigation of biomarkers of the Baobaoqu by LEfSe analysis, with an LDA (Linear Discriminant Analysis) score exceeding 3 as biomarker microorganisms (E).

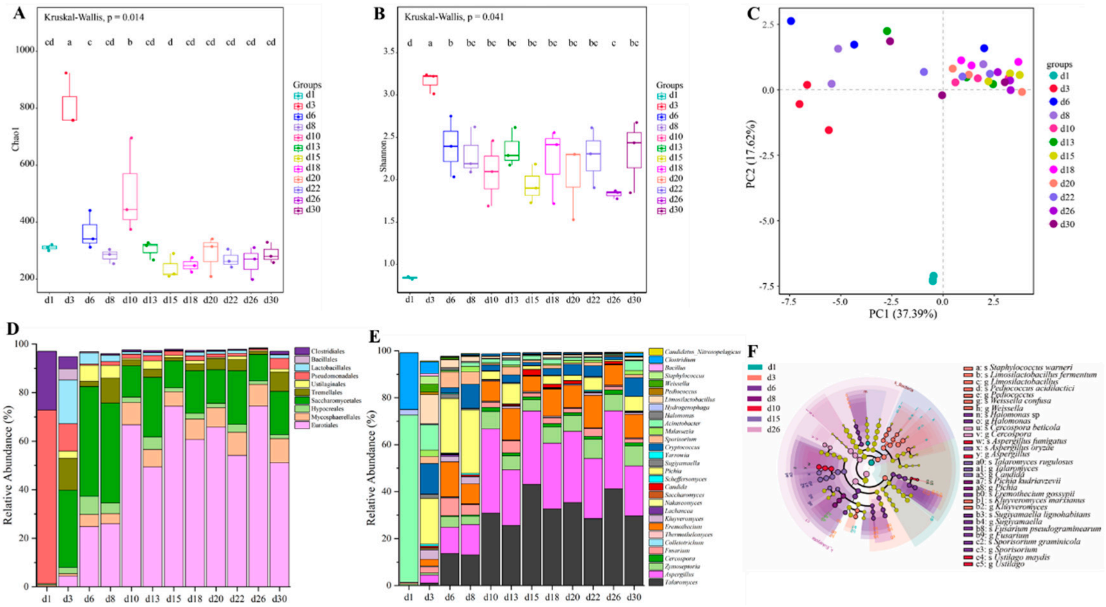

Figure S2. The alpha-diversity indices (Chao1 and Shannon) analysis on the active microbial species richness and diversity by metatranscriptomics analysis (A and B). The principle component analysis (C). Variation of the active microorganisms at orders (D) and genera (E) level in the fermentation process by metatranscriptomics analysis. The investigation of biomarkers of the Baobaoqu by LEfSe analysis, with an LDA score exceeding 3 as biomarker microorganisms (F).

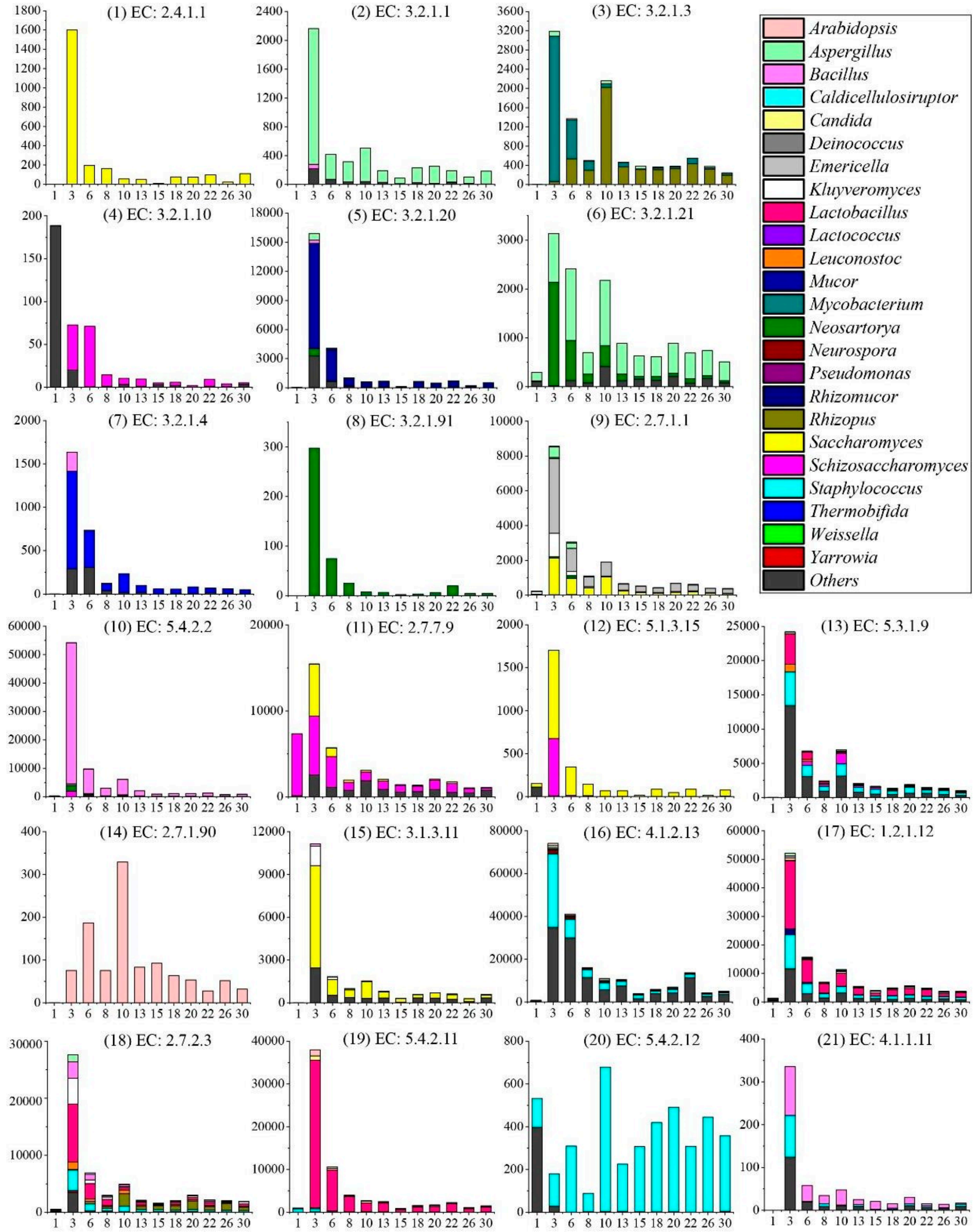
(1) EC: 2.4.1.1
(2) EC: 3.2.1.1
(3) EC: 3.2.1.3
(4) EC: 3.2.1.10
(5) EC: 3.2.1.20
(6) EC: 3.2.1.21
(7) EC: 3.2.1.4
(8) EC: 3.2.1.91
(9) EC: 2.7.1.1
(10) EC: 5.4.2.2
(11) EC: 2.7.7.9
(12) EC: 5.1.3.15
(13) EC: 5.3.1.9
(14) EC: 2.7.1.90
(15) EC: 3.1.3.11
(16) EC: 4.1.2.13
(17) EC: 1.2.1.12
(18) EC: 2.7.2.3
(19) EC: 5.4.2.11
(20) EC: 5.4.2.12
(21) EC: 4.1.1.11
Arabidopsis
Aspergillus
Bacillus
Caldicellulosiruptor
Candida
Deinococcus
Emericella
Kluyveromyces
Lactobacillus
Lactococcus
Leuconostoc
Mucor
Mycobacterium
Neosartorya
Neurospora
Pseudomonas
Rhizomucor
Rhizopus
Saccharomyces
Schizosaccharomyces
Staphylococcus
Thermobifida
Weissella
Yarrowia
Others

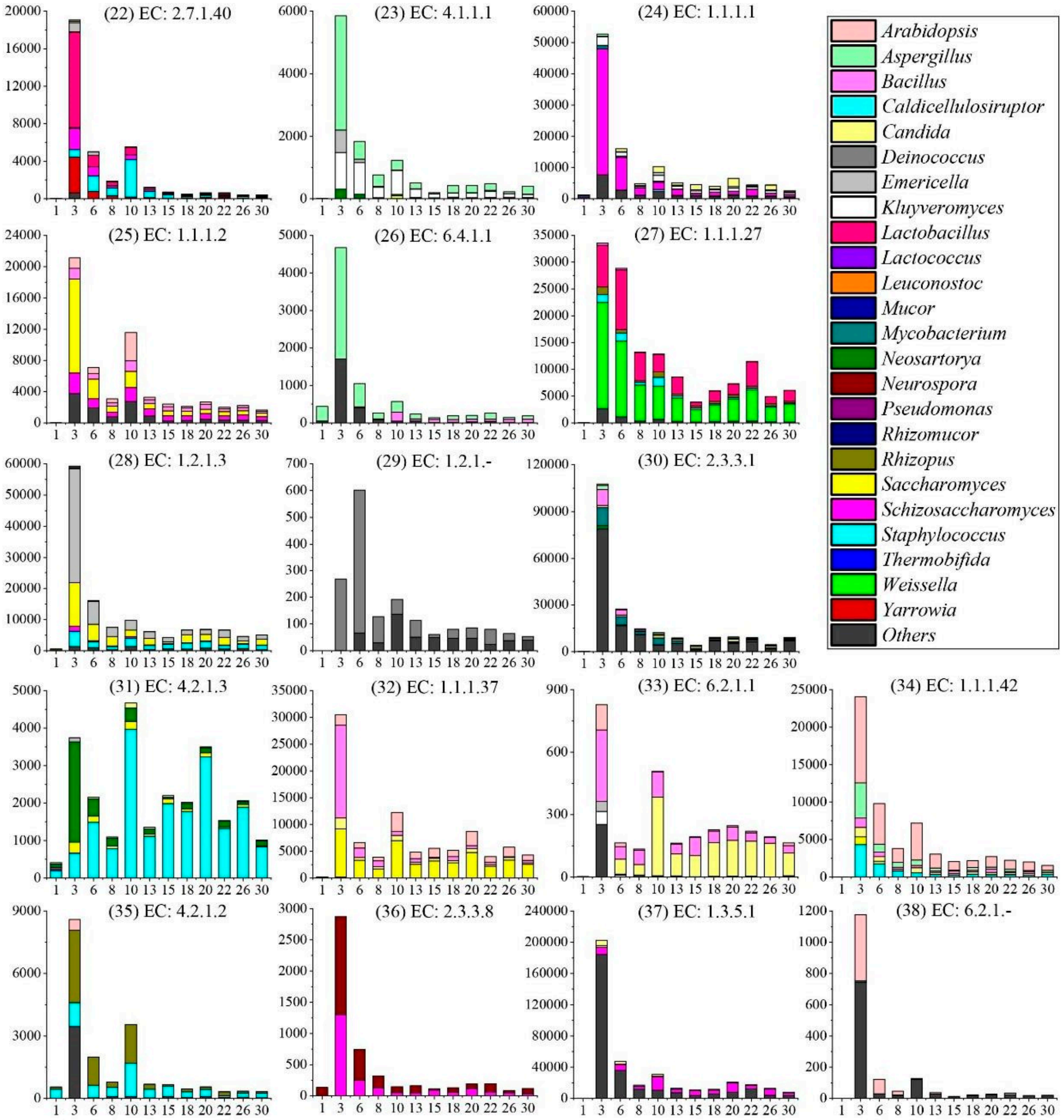
(22) EC: 2.7.1.40
(23) EC: 4.1.1.1
(24) EC: 1.1.1.1
(25) EC: 1.1.1.2
(26) EC: 6.4.1.1
(27) EC: 1.1.1.27
(28) EC: 1.2.1.3
(29) EC: 1.2.1.-
(30) EC: 2.3.3.1
(31) EC: 4.2.1.3
(32) EC: 1.1.1.37
(33) EC: 6.2.1.1
(34) EC: 1.1.1.42
(35) EC: 4.2.1.2
(36) EC: 2.3.3.8
(37) EC: 1.3.5.1
(38) EC: 6.2.1.-
1 3 6 8 10 13 15 18 20 22 26 30
Arabidopsis
Aspergillus
Bacillus
Caldicellulosiruptor
Candida
Deinococcus
Emericella
Kluyveromyces
Lactobacillus
Lactococcus
Leuconostoc
Mucor
Mycobacterium
Neosartorya
Neurospora
Pseudomonas
Rhizomucor
Rhizopus
Saccharomyces
Schizosaccharomyces
Staphylococcus
Thermobifida
Weissella
Yarrowia
Others

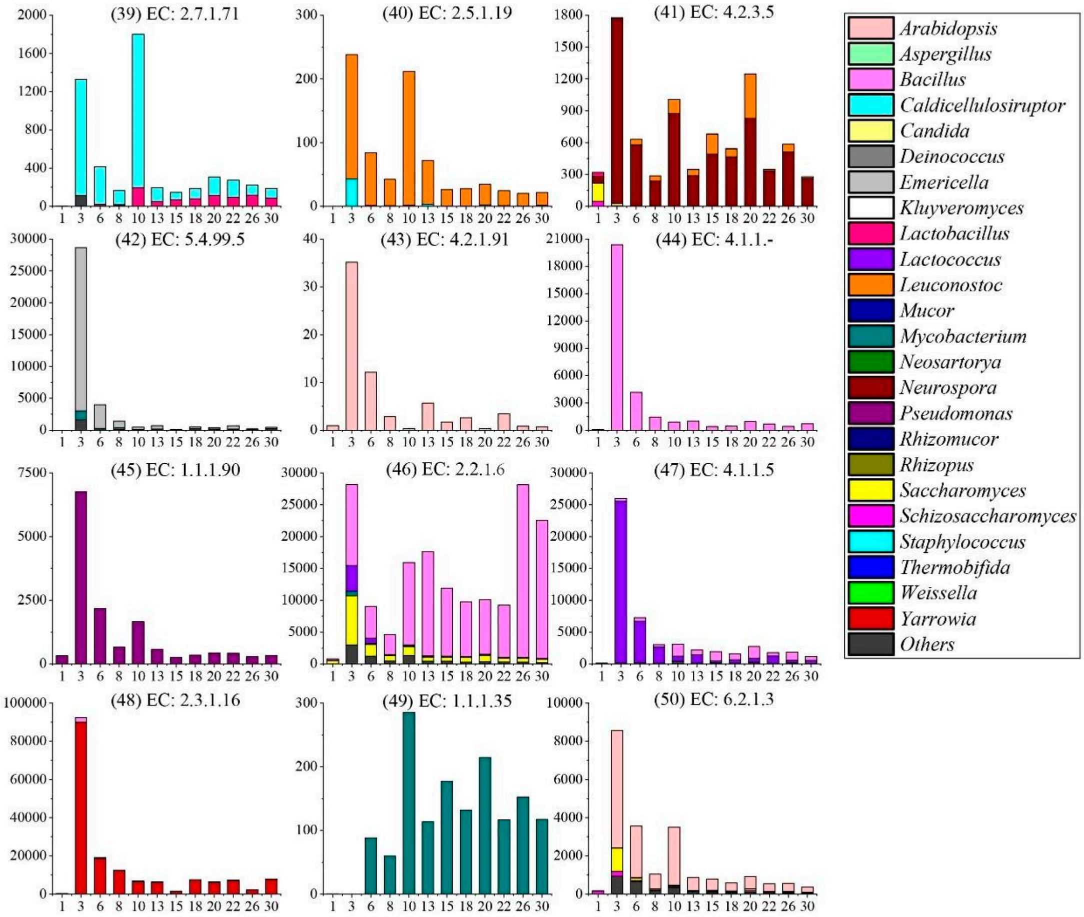

Figure S3. Microbial sources (at the genus level) of key enzymes involved in the reconstructed microbial metabolic network in Fig. 5. The Y axis depicts TPM abundances.

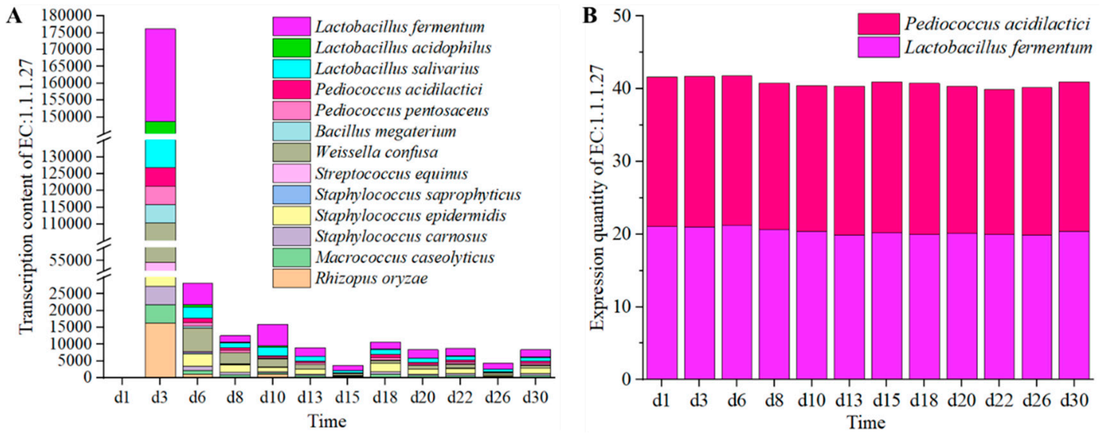

Figurer S4. Distribution map of active microorganisms derived from lactate dehydrogenase by by metatranscriptomics analysis (A). Microbial expression derived from lactate dehydrogenases by Metaproteomics analysis (B).

Table S1. Summary of volatile compounds of Baobaoqu samples (ppm).

Compound name	Class	d1	d3	d6	d8	d10	d13	d15	d18	d20	d22	d26	d30
(R,R)2,3-butanediol	Alcohols	0	0.26851782	0.3555651	0	0	0	0	0.02025586	0.25711387	0.1942548	0.49293752	0.19130388
(S,S)2,3-butanediol	Alcohols	0	0.28521199	0.29323222	0.38359291	0.38153516	0.33406261	0.04824193	0.18904599	0.08151402	0.05794301	0.51854047	0.26676478
1,2-Propanediol	Alcohols	0	0	0	0	0	0	0	0	0	0	0.05274838	0
1-octen-3-ol	Alcohols	0	0	0	0.27646911	0.03239552	0.01882815	0.00629131	0	0	0	0	0
2-Butanol	Alcohols	0	0.12243918	0.04796523	0.01042099	0	0	0	0	0	0	0	0
2-Methylbutanol	Alcohols	0	0	0.53604765	0.43472623	0.18391493	0.01365349	0.00312484	0	0	0	0	0
3-Methyl-2-buten-1 alcohol	Alcohols	0	0.14443889	0.00424538	0.10786127	0.00718862	0.01307416	0.01980601	0	0	0	0	0
3-Methylbutanol	Alcohols	0	0.06861672	0.0866338	0.0440907	0.21459674	0.19135954	0.04037782	0	0	0	0	0
amyl alcohol	Alcohols	0	0	0	0	0	0	0	0.02807297	0	0.03569355	0.10174362	0
amyl alcohol	Alcohols	0	0.0736734	0.09955321	0.05187111	0.03182577	0.01349847	0.00933958	0	0	0	0	0.02634738
benzyl alcohol	Alcohols	0	0	0.10938232	0.03737744	0.16399689	0.17798654	0.07175622	0.02858563	0.04657199	0.06316254	0	0
Capric-2-methylbutyl ester	Alcohols	0	0	0.01440807	1.44799089	0	0	0	0	0	0	0	0

Cetyl alcohol	Alcohols	0	0	0.06986863	0.01105501	0.04067263	0.03007422	0.02131846	0	0	0	0	0
Dodecyl alcohol	Alcohols	0	0	0.01651034	0.9524166	0	0	0	0	0	0	0	0
Furanmethanol	Alcohols	0	0	0	0	0	0	0	0	0	0	0.0293504	0
Furfuryl alcohol	Alcohols	0	0.15425926	0.44626772	2.22875894	0.24261927	0.14042363	0	0.01856064	0.02202852	0.02610348	0	0
Hexanol	Alcohols	0.03158962	0.12220746	0.14576696	0.09566001	0.04705187	0.02283011	0.01598269	0	0	0	0.0317216	0
Phenylethanol	Alcohols	0.02078878	0.41102476	0.66409679	0.15633308	1.3646174	1.30677099	0.84218461	0.54501294	0.5594067	0.48942979	0.33131746	0.47344704
propanol	Alcohols	0	0.38911391	0.16746498	0	0	0	0	0	0	0	0	0
(E,E)-2,4-decadienal	Aldehydes and ketones	0	0	0.03353938	0.40291513	0.02173468	0	0	0	0	0	0	0
2,5-Dimethylbenzaldehyde	Aldehydes and ketones	0	0	0	0	0	0	0	0	0	0	0	0.02380218
2-Nonanone	Aldehydes and ketones	0	0.12617328	0.01264942	0.0209157	0	0	0	0	0	0	0	0
3-hydroxy-2-butanone	Aldehydes and ketones	0	0.03656889	0.07313115	0.0401667	0.04699126	0.03218168	0.01655428	0	0	0	0	0
benzaldehyde	Aldehydes and ketones	0	0	0	0.03435613	0.25127011	0.16384751	0.11071193	0	0	0	0	0
Ethyl 2-methylbutyrate	Esters	0	0.69612822	0	0.06674182	0.04537616	0	0	0	0	0	0	0

Ethyl 3-hydroxy-butyrate	Esters	0	0.03496487	0.04247419	0.15522124	0.02056467	0.01231804	0.01919655	0	0	0	0	0
Ethyl 4-methyl-2-hydroxyvalerate	Esters	0	0	0.04647555	0	0	0	0.34670149	0	0	0	0	0
Ethyl 9-hexadecenoate	Esters	0.05597317	0	0.0256313	0.08491731	0.02127718	0.02314882	0.01397452	0.01573506	0.00938993	0	0	0
Ethyl butyrate	Esters	0	0	0	0.05607343	0.04346416	0	0	0	0	0	0	0
Ethyl caprate	Esters	0.55086221	0	0.14240822	0.31560178	0.04216994	0.03575402	0.03219221	0	0	0	0	0
Ethyl caproate	Esters	0	0.06795174	0.31474886	0.5732306	0.36006004	0.16532142	0.05058504	0	0.31884692	0.06877765	0.18536966	0.07999478
Ethyl caprylate	Esters	0	0	0.0511016	0.12271732	0.0482321	0.03368657	0.01479139	0.01467149	0.03186986	0.01802624	0.03822805	0.02676593
Ethyl dodecanoate	Esters	0.23960038	0	0.63973034	0.03404564	0.28295916	0.1038198	0.10517549	0.04298503	0	0	0	0
Ethyl enanthate	Esters	0	0	0	0	0	0	0	0	0.02010605	0.01161956	0	0.01647733
Ethyl hexadecanate	Esters	0.07958216	0	0.0893625	0.03115039	0.12605179	0.27637555	0.13123051	0.24323259	0.29141919	0.16947198	0.10754937	0.16410443
Ethyl lactate	Esters	0	0.21683547	0.35660865	0.10903562	0.03684068	0.01816963	0.01434398	0	0	0	0	0
Ethyl nonanoate	Esters	0	0	0.02189479	0	0	0	0	0.03026615	0.06676187	0.03500126	0	0.03679687
Ethyl oleate	Esters	0.01450258	0	0	0.03807839	0.01279728	0.04372139	0.01639971	0.04352558	0.07620525	0.0343573	0.0237658	0.03287689
Ethyl tetradecanoate	Esters	0.02245425	0	0.03337037	0.04949937	0.03043653	0.03871145	0.02674676	0.02965677	0.0399946	0.02453161	0	0.02570176

Methyl benzoate	Esters	0	0	0	0	0	0	0	0.02905724	0.01609985	0	0	0
Phenylethyl acetate	Esters	0.01966433	0	0.04746136	0.02985622	0.01323877	0.00703778	0.00232538	0	0	0	0	0
γ-Nonolactone	Esters	0	0	0.01789869	0.05750498	0.00780944	0	0	0.01896139	0	0	0	0
acetic acid	Organic acids	0	3.44935241	2.43005253	0.04498418	0.14739282	0.06502931	0.01581698	0	0	0	0.06340689	0
Hexanoic acid	Organic acids	0	0	0.10201505	0	0	0	0	0	0	0	0	0
Trans-13-eicosenoic acid	Organic acids	0	0	0	0	0	0	0	0	0	0	0	0.0132888
1,2,4-Trimethoxybenzene	Others	0	0	0	0	0	0.02604121	0.01529589	0	0.00738633	0	0	0
1,2-Methoxy-4-vinylbenzene	Others	0	0	0	0	0	0	0	0.0222218	0.01287405	0	0	0
2,3-Dihydrobenzofuran	Others	0	0	0	0	0	0	0	0.01292473	0	0	0	0
2-acetylpyrrole	Others	0	0	0	0	0	0	0	0	0.01357782	0	0	0
2-Amylfuran	Others	0.02714301	0.19098883	0.19582536	0.45354238	0.36762638	0.15748001	0.01143518	0	0	0	0	0
2-naphthol	Others	0	0	0	0	0.02224128	0.01878424	0.009304	0	0	0	0	0
3-Phenylfuran	Others	0	0	0	0	0	0	0	0	0.00828783	0	0	0
4-Vinylguaiacol	Others	0	0	0.03036278	0.14694225	0.01945657	0.02489069	0.01696232	0	0	0.01075571	0	0

furan	Others	0	0	0.11869825	0.69088992	0.07690927	0	0	0	0	0	0	0
2,3-Dimethylpyrazine	Pyrazines	0	0	0	0	0	0	0	0.01059764	0	0	0.02356245	0
2,6-Dimethylpyrazine	Pyrazines	0	0	0	0	0	0.0120838	0.02596908	0.02129144	0.02109428	0.02369167	0.05453449	0.02641809
2-Ethyl-3,5-dimethylpyrazine	Pyrazines	0	0	0	0	0	0	0	0	0	0	0	0.01403982
2-ethyl-6-methylpyrazine	Pyrazines	0	0	0	0	0	0	0	0.01712668	0	0	0	0
2-Vinyl-6-methyl-pyrazine	Pyrazines	0	0	0	0	0	0	0	0	0.01657514	0	0	0
Trimethylpyrazine	Pyrazines	0	0	0	0	0	0.04003166	0.06975452	0.02425081	0.03722763	0.03226962	0.07611465	0.04108584

Table S2. Raw and clean data statistics of Baobaoqu samples by metagenomics.

Samples	Raw reads	Raw data (bp)	GC (%)	Q20 (%)	Q30 (%)	Clean reads	Clean data (bp)	Clean data (%)
d1-1	67750148	10162522200	45.78	94.47	86.53	62228350	9334252500	91.85
d1-2	71395908	10709386200	45.61	93.44	84.48	64827756	9724163400	90.80
d1-3	66403768	9960565200	45.73	93.54	84.61	60253114	9037967100	90.74
d3-1	76480000	11472000000	46.19	94.16	86.04	66875588	10031338200	87.44
d3-2	66284886	9942732900	43.26	93.99	85.63	59667874	8950181100	90.02
d3-3	75120000	11268000000	44.03	94.05	85.38	66942988	10041448200	89.11
d6-1	78720000	11808000000	42.34	95.03	87.29	66957574	10043636100	85.06
d6-2	74880000	11232000000	45.48	98.79	95.18	66892694	10033904100	89.33
d6-3	72880000	10932000000	45.28	98.64	94.59	66877068	10031560200	91.76
d8-1	75200000	11280000000	42.80	98.61	94.57	66905660	10035849000	88.97
d8-2	81280000	12192000000	43.80	95.52	88.72	66847282	10027092300	82.24
d8-3	77920000	11688000000	42.91	94.92	87.23	66905838	10035875700	85.86
d10-1	79360000	11904000000	44.11	98.36	94.77	66742048	10011307200	84.10
d10-2	67543978	10131596700	43.02	98.19	94.00	59903504	8985525600	88.69
d10-3	73280000	10992000000	38.83	98.57	94.12	66869042	10030356300	91.25
d13-1	72560000	10884000000	38.74	98.48	94.57	66757776	10013666400	92.00
d13-2	75120000	11268000000	42.74	97.95	93.91	66807906	10021185900	88.93
d13-3	74720000	11208000000	40.15	98.14	93.86	66810194	10021529100	89.41
d15-1	77200000	11580000000	41.17	94.59	86.28	67056926	10058538900	86.86
d15-2	83760000	12564000000	41.33	94.6	85.87	66928656	10039298400	79.91
d15-3	81360000	12204000000	41.95	95.01	87.07	66885608	10032841200	82.21

d18-1	81040000	12156000000	41.51	94.86	86.68	66959112	10043866800	82.62
d18-2	77920000	11688000000	40.35	94.24	85.54	66740076	10011011400	85.65
d18-3	79760000	11964000000	39.52	94.80	86.81	67056490	10058473500	84.07
d20-1	80160000	12024000000	39.82	95.20	87.34	66973066	10045959900	83.55
d20-2	78400000	11760000000	42.37	98.71	94.70	66745380	10011807000	85.13
d20-3	82880000	12432000000	45.63	94.89	87.38	66923616	10038542400	80.75
d22-1	75040000	11256000000	40.06	98.34	94.17	66848070	10027210500	89.08
d22-2	76640000	11496000000	39.64	94.91	86.91	66922840	10038426000	87.32
d22-3	81280000	12192000000	44.04	94.75	87.05	66847552	10027132800	82.24
d26-1	77680000	11652000000	41.96	98.65	94.43	66868066	10030209900	86.08
d26-2	77840000	11676000000	40.34	94.92	87.66	67024670	10053700500	86.11
d26-3	78720000	11808000000	42.01	95.20	87.01	66878786	10031817900	84.96
d30-1	82880000	12432000000	41.12	95.62	88.44	66933712	10040056800	80.76
d30-2	81920000	12288000000	40.25	95.24	87.82	66970648	10045597200	81.75
d30-3	86960000	13044000000	40.14	95.56	88.30	67028484	10054272600	77.08

Table S3. Assembly quality statistics of Baobaoqu samples by metagenomics.

Samples	Min sequence length (bp)	Max sequence length (bp)	Contig number	N50 (bp)	N90 (bp)	Total sequence length (bp)
d1-1	300	35147	438330	512	350	219613798
d1-2	300	35147	431764	513	351	217943005
d1-3	300	35147	368412	514	352	185627200
d3-1	300	633201	85763	1363	399	81451531
d3-2	300	495699	77251	1743	400	83935394
d3-3	300	495699	82663	1822	418	93849867
d6-1	300	640132	84977	1709	475	103432525
d6-2	300	389147	61207	2142	468	78222351
d6-3	300	633201	66025	1997	437	78283691
d8-1	300	495699	122240	1716	447	140961241
d8-2	300	495699	77667	2044	448	96554561
d8-3	300	577454	73790	1818	444	88684339
d10-1	300	576267	185049	2349	466	237811074
d10-2	300	576137	149220	2908	518	224990481
d10-3	300	640132	135801	1656	435	153794535
d13-1	300	576267	149989	2788	507	220749710
d13-2	300	495699	183434	2447	425	228403423
d13-3	300	495699	192999	2154	453	242864559
d15-1	300	495699	139568	2355	520	200478167
d15-2	300	377210	151398	1783	463	179353308

d15-3	300	495699	141701	2306	523	202033078
d18-1	300	495699	145499	2405	486	195841275
d18-2	300	495699	145570	2991	500	215560538
d18-3	300	495699	139696	2420	495	192322782
d20-1	300	576267	122756	2578	547	183974618
d20-2	300	576267	147431	4310	528	242900541
d20-3	300	526656	96034	4571	437	124538596
d22-1	300	577454	134936	4129	540	222220460
d22-2	300	495699	137260	2161	548	195501556
d22-3	300	396380	105030	5586	572	201013646
d26-1	300	576267	125811	2665	492	179496118
d26-2	300	495699	123349	1635	397	130152132
d26-3	300	576267	125512	4369	546	213681127
d30-1	300	396380	151680	1807	484	186022416
d30-2	300	495699	123795	1719	459	146755113
d30-3	300	640132	104318	2416	536	152338125

Table S4. Raw, clean data, and assembly quality statistics of Baobaoqu samples by metatranscriptomics.

Samples	Contig Number	Assembly Length (bp)	N50 (bp)	N90 (bp)	Max (bp)	Min (bp)	Clean reads (%)	Clean data (bp)	Clean data (%)	Raw base	Clean base
d1-1	24668	12759912	511	328	7795	300	99.81	9199819600	85.99	10699027800	10469704682
d1-2	26939	14077499	519	328	7542	300	99.79	9271591400	86.66	10699027800	10467975686
d1-3	24600	12797282	516	328	13168	300	99.75	9278478400	86.72	10699027800	10447836310
d3-1	54718	46612192	1119	385	22974	300	99.81	9999327800	93.46	10699027800	10548226432
d3-2	51664	38992595	925	357	21471	300	99.88	9854840400	92.11	10699027800	10567848180
d3-3	64006	53707358	1099	379	23897	300	99.88	9981338000	93.29	10699027800	10562377362
d6-1	29489	22867034	954	369	22954	300	99.92	9998650400	93.45	10699027800	10551782496
d6-2	32094	26480514	1051	384	13723	300	99.94	9710698400	90.76	10699027800	10586735948
d6-3	22726	17678010	957	374	12374	300	99.93	10015342200	93.61	10699027800	10580269316
d8-1	29332	21800679	881	369	13572	300	99.92	9966324200	93.15	10699027800	10560457978
d8-2	17640	10683785	640	342	11820	300	99.89	9865861600	92.21	10699027800	10509652824
d8-3	27087	16603406	641	343	15196	300	99.91	9888539400	92.42	10699027800	10550888146
d10-1	31083	26217335	1146	372	16090	300	99.80	9864432600	92.20	10699027800	10505802716
d10-2	24073	19595671	1053	378	16077	300	99.83	9779708200	91.41	10699027800	10529206554
d10-3	20512	22136473	1733	424	27344	300	99.72	9976055200	93.24	10699027800	10480290420
d13-1	16178	9461217	605	339	8765	300	99.25	9587264600	89.61	10699027800	10106746154
d13-2	20658	13801002	737	350	13571	300	99.70	9742445000	91.06	10699027800	10432775448
d13-3	17656	15935408	1273	394	14925	300	99.79	9622275000	89.94	10699027800	10481488352
d15-1	19066	14469488	917	371	12751	300	99.80	9884084400	92.38	10699027800	10429469712

d15-2	19527	14233673	849	366	12487	300	99.80	9834700000	91.92	10699027800	10447661528
d15-3	15807	11179483	819	358	12269	300	99.88	9556478000	89.32	10699027800	10511732734
d18-1	14138	8608270	632	338	11726	300	99.31	9586807600	89.60	10699027800	10177427692
d18-2	21187	17637801	1065	386	13247	300	99.83	9971222200	93.20	10699027800	10489305336
d18-3	15793	9963850	674	343	7320	300	99.88	9623374000	89.95	10699027800	10518673152
d20-1	19857	15607319	981	369	14705	300	99.71	9665327000	90.34	10699027800	10435397858
d20-2	22672	16983744	897	361	14157	300	99.79	9773748000	91.35	10699027800	10498007110
d20-3	18202	14952906	1039	383	13354	300	99.79	10167075400	95.03	10699027800	10499560374
d22-1	20014	14181809	809	360	12148	300	99.78	9826236400	91.84	10699027800	10472961878
d22-2	20484	12223758	616	339	12444	300	99.85	9697381400	90.64	10699027800	10536278894
d22-3	24604	17874436	842	359	17323	300	99.79	9681528400	90.49	10699027800	10474316870
d26-1	20231	13976272	782	355	8563	300	98.66	9985884600	91.21	10947842400	10212218770
d26-2	18571	13526967	849	366	11369	300	99.67	9768911600	91.31	10699027800	10422862208
d26-3	12846	9780739	921	365	9558	300	99.78	9713210800	90.79	10699027800	10482759894
d30-1	9735	5740751	598	335	9703	300	99.73	9516434400	88.95	10699027800	10406491460
d30-2	16027	13324065	1078	382	9102	300	99.68	9719734200	90.85	10699027800	10415158334
d30-3	10852	6550829	627	340	12464	300	99.72	9763875200	91.26	10699027800	10416590242
